# A randomized controlled trial of Scanning Eye trAining as a Rehabilitation Choice for Hemianopia after stroke (SEARCH)

**DOI:** 10.1177/17474930251330140

**Published:** 2025-03-13

**Authors:** Fiona J Rowe, Ella Brayshaw, Michaela Brown, Kausik Chatterjee, Avril Drummond, Christine Hazelton, Brin Helliwell, Lauren R Hepworth, Claire Howard, Stevie Johnson, Carmel Noonan, Catherine Sackley, Laura Wright

**Affiliations:** 1 Institute of Population Health, University of Liverpool, Liverpool, UK; 2 University of Liverpool, Liverpool, UK; 3 Countess of Chester NHS Foundation Trust, Chester, UK; 4 University of Nottingham, Nottingham, UK; 5 Glasgow Caledonian University, Glasgow, UK; 6 Patient and Public Representative, UK; 7 Northern Care Alliance NHS Foundation Trust, Salford, UK; 8 Royal National Institute of Blind People, London, UK; 9 Liverpool University Hospitals NHS Foundation Trust, Liverpool, UK

**Keywords:** Homonymous hemianopia, visual scanning training, randomised controlled trial, sham training, stroke, quality of life

## Abstract

**Background:**

Hemianopia is common after stroke. We aimed to evaluate clinical effectiveness of visual scanning training (VST) versus sham training, for homonymous hemianopia.

**Design:**

Randomized controlled, parallel, double-blind, two-arm trial.

**Methods:**

Prospective, multicentre randomized controlled trial (RCT) with 34 UK stroke units.

**Participants:**

Adult stroke survivors with confirmed stable homonymous hemianopia.

**Inclusion criteria:**

Clinically diagnosed stroke, 18+ years, stable hemianopia, >4 weeks and <26 weeks post-stroke onset, able to engage in training, informed/proxy consent.

**Interventions:**

Arm A (VST) or arm B (sham training) for minimum 30 minutes, 7 days per week over 6 weeks. Follow-up to 26 weeks.

**Objective:**

Evaluate clinical effectiveness of VST versus sham training, for homonymous hemianopia.

**Outcomes:**

Primary outcome measurement was change in the National Eye Institute Visual Function Questionnaire 25 (NEI VFQ-25) score from baseline to 26 weeks. Secondary outcome measurements were change in the Nottingham Extended Activities of Daily Living (NEADL), EuroQoL (EQ-5D-5L), Brain Injury-Related Visual Impairment—Impact Questionnaire (BIVI-IQ), visual field measurement (Esterman program), visual scanning performance, and adverse events from baseline to 26 weeks.

**Randomization:**

Web-based randomization system stratified by partial/complete hemianopia.

**Blinding:**

Participants and primary outcome assessor blinded to treatment allocation.

**Results:**

In total, 161 participants were randomized; 80 to the VST group and 81 to the sham group. One participant was randomized in error and two withdrew consent to use data so were not included. Of 158 participants, 78 were in the VST group and 80 in the sham group. No participants were unblinded. All participants began their training allocation. During the first 6 weeks of training, 56 (72%) and 58 (73%) undertook training every day or most days in the VST and sham groups, respectively. There were 37 withdrawals from the trial: 18 in the VST group and 19 in the sham group. Both groups were comparable in terms of baseline characteristics. Primary analysis of covariance (ANCOVA) analysis was carried out on 104 participants with VFQ-25 data at both baseline and 26 weeks; sensitivity analysis was undertaken for 120 participants. Estimated mean difference at 26 weeks, adjusting for baseline score and hemianopia type was −4.04 (95% confidence interval (CI): –9.45 to 1.36; p = 0.141) for primary analysis and −2.33 (95% CI: −7.42 to 2.75; p = 0.365) for sensitivity analysis. There were no significant differences between groups for primary and secondary outcome measure comparisons from baseline to 26 weeks. Adverse events, reported for 20 participants, included eye strain, headache, and blurred vision.

**Conclusion:**

Both groups improved in all primary and secondary outcomes. There were no differences between the groups for any outcome measure. However, it was possible that there was a placebo effect from additional information resources and clinician input during the first 6 weeks of treatment and for the sham training providing a treatment effect. These aspects warrant further research.

## Introduction

Sight is often regarded as our most critical human sense playing a central role in daily life and overall quality of living.^1^ A crucial component of sight is the field of vision. Homonymous hemianopia is characterized by loss of the field of vision on one side of the visual field in both eyes.^2^ This sudden impairment disrupts a person’s ability to “see” half of their visual world with devastating consequences to their quality of life.

Hemianopia is reported in about 30% of stroke survivors.^3–5^ Recovery rates vary, with only 15% experiencing full recovery within 2–3 weeks, 35% achieving partial recovery and 50% showing no recovery at all.^6^ Despite its prevalence, the management and rehabilitation of hemianopia remain inconsistent. Issues such as limited access to vision screening, delayed referrals, and the lack of standardized treatment protocols compound the challenges faced by these individuals.^7^

The impact of hemianopia is devastating to individuals with considerable consequences for daily life. Patients with stroke who have hemianopia show substantial reductions in both health- and vision-related quality of life.^8^ Hemianopia on admission is linked to poor early survival and can lead to an increased risk of falling,^2^ impaired ability to mobilize, low mood, and higher levels of institutionalization, with further impacts on social functioning and emotional well-being.^9–11^ The risk of depression is three times greater in people with sight loss than those with good sight.^8^ Visual field loss reduces the person’s abilities to participate in rehabilitation, ultimately resulting in poorer long-term recovery. Extensive field loss exacerbates these issues, leading to reading difficulties, driving restrictions, and social isolation. Addressing these challenges through targeted rehabilitation is essential to improve visual function, enhance independence, and reduce the risk of accidents and social disengagement.

However, there is limited evidence that compensatory visual search training is effective at improving search scanning outcomes.^12^ Improving search is essential to improving speed and accuracy of detection of objects to the affected, blind side. Improvement in speed and accuracy of blind-side object detection could imply improved adaptation to hemianopia.^13^ Adaptation is key to acquiring better visual function and, in turn, could lead to improvements in other domains such as increased confidence in mobility/navigation and consequent increased independence.^14^ The Cochrane review recommended visual scanning/search training (VST) as having potential but requiring high-quality randomized controlled trials (RCTs) to compare search interventions with placebo (sham training), no treatment or usual care.^12^

The VISION pilot trial^15^ investigated the feasibility of a novel paper-based VST compared to substitutive Fresnel prisms or standard care (no treatment). Paper-based VST addressed accessibility challenges faced by stroke survivors, particularly those with limited computer literacy or access.^15^ Accessibility is of importance to ensure equitable availability and use of training regardless of setting. The results indicated that the portable paper-based VST (self-administered at home without direct clinical support) was easily accessible and could be widely implemented immediately on diagnosis of hemianopia early after stroke onset—facilitating early rehabilitation. Furthermore, the trial provided information on choice of outcome measure and informed sample size calculation for a follow-on trial of VST versus sham training—the SEARCH (Scanning Eye trAining as a Rehabilitation Choice for Hemianopia after stroke) trial. The primary outcome measure was the National Eye Institute Visual Function Questionnaire 25 (NEI VFQ-25) questionnaire. It was hypothesized that improvements would occur in both treatment arms, but the addition of VST would be more effective than standard care. We now report the results of the SEARCH trial; the aim of which was to determine the clinical effectiveness of delivering paper-based VST compared to sham training.

## Methods

### Trial design

SEARCH was a randomized controlled multicentre trial with National Health Service (NHS) research ethical approval (21/WA/0030). The detailed trial protocol is reported elsewhere (www.searchtrial.co.uk) but essentially was a prospective, parallel 2-arm trial with 1:1 allocation comparing VST to sham training. For reporting the trial results, we followed the CONSORT, CONSORT-PRO, and TIDieR guidelines.^16–18^

### Participants

Participants were eligible for inclusion if they met the following criteria:

- Clinically diagnosed stroke,
- 18+ years,
- Stable (non-changing) hemianopia,
- Greater than 4 weeks and less than 26 weeks from stroke onset,
- Able to engage in training,
- Informed/proxy consent,
- Written and informed consent obtained and agreement to comply with the requirements of the study from participant.

Participants were not eligible for inclusion if:

- Presence of severe visual inattention (assessed by stroke team using cancelation task),
- Other documented serious concomitant medical condition (e.g. life expectancy <6 months).

Participants were recruited from stroke units based in 34 UK NHS hospitals with integrated stroke and orthoptic services. Recruitment was through inpatient or outpatient settings dependent on length of hospital stay for the individual stroke survivor. Potentially eligible participants were identified by stroke research nurses and screened for inclusion by a local principal investigator (PI: a qualified orthoptist registered with the Health and Care Professions Council, UK). Participants eligible for inclusion, and providing consent, attended a baseline orthoptic assessment (typically undertaken in the eye clinic) which included assessment and documentation of patient demographics, visual signs and symptoms, visual acuity and visual field measures, any additional ocular problems, comorbidity, severity of stroke, and level of disability.

### Recruitment and randomization

Participants were individually randomized to one of two treatment groups using a secure 24-hour web-based randomization program to ensure allocation concealment. Randomization lists were generated by an independent statistician at the Liverpool Clinical Trials center (not otherwise involved in the SEARCH trial) using random variable block sizes stratified by extent of hemianopia (partial or complete) with treatment allocation ratio of 1:1. The local PI obtained the treatment allocation and subsequently assigned the participant to the indicated treatment arm.

#### Blinding

The participants recruited to the trial were blinded/masked to treatment allocation. Treating clinicians were aware of allocation by the type of the treatment provided. The primary outcome assessor at the University of Liverpool was blinded to treatment allocation.

### Interventions

#### Arm A: VST

The SEARCH trial used the VISION intervention—a paper-based VST package, consisting of training sheet and training instructions. It is portable, self-administered, does not need expert set-up, does not require expertise to use it or support its use, and can be used at home or any care setting.

The sheet consists of A4-size landscape paper (Supplementary Figure 1) with targets filling the paper.^15^

#### Arm B—sham training

The control was sham training comprising a series of slow, tracking eye movements undertaken with both eyes open (versions) and with each eye covered in turn (ductions). These movement patterns did not engage scanning eye movements. A training sheet and instructions were provided, similar to the intervention arm.

#### Dosage and administration

*For both intervention and sham training*: Participants were instructed to train for at least 30 minutes for 7 days/week over 6 weeks (minimum plateau “dose” of 20 hours^19^), and record this in a diary. The intervention could be undertaken in any location and completed individually. The intervention and sham training were provided by the research orthoptist following the baseline assessment and consent process. This included demonstration of the training, however, no specific training was required for administering the training. The instructions provided were self-explanatory and co-written with patient and public involvement.

Aphasia-friendly, video or audio versions of the written instruction sheets were available, to maximize accessibility in a visually impaired population and encourage recruitment of patients with mild cognitive or communication impairments.

#### Treatment modifications

Should the patient not manage the treatment dose indicated during the first 6 weeks of treatment, the treatment period could be extended such that the patient subsequently achieved the minimum treatment dose of 20 hours. The patient could opt to do either 30 minutes continuous training per day or to cumulate daily amounts, e.g. two ×15 minutes training each day, or three ×10 minutes training each day, in order to manage fatigue, eye strain and/or headaches.

#### Assessment of compliance

Participants received weekly telephone contact from clinical staff for support and encouragement during the first 6 weeks. Weekly calls encouraged:

- Adherence to treatment, ensuring completion of patient diaries (time using the intervention/documenting views on use of the intervention) [intervention arm].
- Provision of information support, checking diary for use of self-compensation/related issues [control arm].
- Provision of reminder to attend follow-up appointments and ensure completion of questionnaires [both arms].

Compliance with the intervention and sham training was taken from participant self-reports in their patient diaries.

### Outcomes

Effectiveness of the trial treatments were measured through the period of the trial using both objective and subjective measures undertaken during eye clinic appointments or at home visits. All end points were measured at 26 weeks after baseline assessment. Participants typically completed their questionnaire booklets before undergoing assessment of outcome measures. If the participant required help with completing the forms (because of difficulties with writing, reading or aphasia), this was recorded by the PI.

The primary outcome measurement was the NEI VFQ-25, a questionnaire designed to measure vision-related activities of daily living.^20^ Primary endpoint was perceived ability relating to activities of daily living at 26 weeks post-baseline assessment.

Secondary outcome measurements included the Nottingham Extended Activities of Daily Living (NEADL) Scale,^21^ EQ-5D-5L,^22^ Brain Injury associated Visual Impairment Impact Questionnaire (BIVI-IQ),^23^ visual field measurement (Esterman programme^15^), visual scanning performance, and adverse events (AEs). Objective visual scanning performance was assessed via a table-top task. Speed of completion (response time) of each task was recorded in minutes: seconds. Accuracy was recorded as the percentage of objects correctly identified at first attempt. Patient-reported AEs were collected from completed patient diaries.

Secondary endpoints were change in functional mobility score, extended daily living index score, health-related quality of life score, degrees of visual field, percentage correct object identification, speed of object identification, from baseline to 26 weeks post-baseline assessment.

### Assessments

Assessments were made at the baseline visit (T0) and at follow-up visits at 6 weeks (T6), 12 weeks (T12), and 26 weeks (T26). Each follow-up visit had a ±2-week window to take into account availability of the participant to attend the follow-up visit. Baseline data included age, sex, ethnicity, stroke type, laterality and severity (Barthel Index), area of brain affected, time since stroke onset, visual symptoms, best-corrected visual acuity at near and distance (logMAR), clock cancelation task, ocular motility, and visual field assessment (confrontation and/or Esterman program). Follow-up was provided by NHS outpatient services or home visits but with the same assessment process regardless of setting.

### Sample size calculation

The primary outcome measure was the NEI VFQ-25 questionnaire. The sample size calculation was based on Borm et al.^24^ and was informed by the VISION trial;^15^

In the pilot trial a mean score difference of 8 points on the NEI VFQ-25 questionnaire was reported (mean score of 60 in the visual scanning arm at baseline and 68.4 at 26 weeks). This difference was clinically/statistically significant, and also reported by Crotty et al.^25^ Thus, based on a pooled standard deviation of 21.2 and correlation between baseline and 26-week score of 0.7 (also reported in the pilot trial) and a two-sided significance level of 0.05, 114 patients would give 80% power to detect this significant difference; increasing to 142 (71 per arm) to allow attrition of 20% from loss to follow-up/death.

### Analysis plan

Primary analysis used the principle of intention to treat, based on all randomized participants, as far as was practically possible. A p value of <0.05 was used to declare statistical significance for all analyses and results are presented with 95% confidence intervals.

The primary outcome (NEI VFQ-25) was analyzed at baseline, 6-, 12-, and 26-week follow-up. The mean and standard deviation of the total score are presented at each time point. We stratified for partial/complete hemianopia but no other factors. The hypothesis of no difference between the two treatment arms at 26-week follow-up was tested using analysis of covariance (ANCOVA), controlling for baseline measurements. Longitudinal analyses explored changes over time in quality of life for both vision- and health-related measures, and changes in objective measurements of visual scanning performance (speed and accuracy results) across groups. All AEs and serious adverse events (SAEs) are presented, identified by treatment group.

## Results

### Screening

Overall, 3869 stroke survivors were screened for eligibility of which 2599 (67.2% were ineligible. Of those that were eligible, a further 110 (2.8%) were not consented and 999 (25.8%) were not randomized for other reasons. Reasons for non-recruitment are outlined in Supplementary Table 1. One hundred and sixty-one participants were randomized to this trial (12.7% of eligible patients).

Recruitment started in May 2021 but was delayed by continuing COVID-impact. To mitigate for slow recruitment, the number of recruitment sites was increased from 25 to 34. Subsequently, to mitigate for higher than anticipated loss to follow-up, the target recruitment number was raised from 142 to 157 (Supplementary Figure 2). Recruitment ended in July 2023 and follow-up ended in March 2024.

#### Study population

Overall, 161 participants were randomized; 80 in the VST group and 81 in the sham training group (Supplementary Figure 3). Three participants withdrew consent to use data so were not included in any analysis. Of 158 participants, 78 were in the VST group and 80 in the sham training group; none were unblinded.

There were 37 withdrawals: 18 in the VST group and 19 in the sham training group. Most were unwilling to dedicate time for follow-up (n = 11) or were lost to follow-up (n = 11). Other reasons included undertaking other scanning training (n = 1), non-related AE (n = 2), death (n = 4), moved out of area (n = 1), other health issues or further stroke (n = 3), no reason given (n = 1), improvement or deterioration in vision (n = 2), and unwilling to continue training (n = 1).

Protocol deviations were equally spread across groups (Supplementary Table 2). Baseline characteristics are outlined in Supplementary Table 3. Supplementary Table 4 outlines baseline visual measurements. The VST and sham training group participants were comparable at baseline.

#### Compliance with treatment

All participants began their training allocation. In total, 37 (23%) participants did not complete a minimum training period of 20 hours; 12 in the VST group and 25 in the sham training group. During the first 6 weeks of training, 56 (71.8%) and 58 (72.6%) undertook training every day or most days in the VST and sham training groups, respectively. Supplementary Table 5 outlines the summary of treatment hours over the first 6 weeks and in total over the follow-up period.

After the required 6-week treatment period, participants could continue if the dose had not been achieved, or participants could continue voluntarily and thus, increase the dose. After 12 weeks, they could also continue voluntarily. At week 12, 47 participants in the VST group and 43 participants in the sham training group reported that they had continued to use treatment. At 26 weeks, 34 participants in the VST group and 24 participants in the sham training group reported that they had continued to use treatment.

#### Primary outcome analysis

ANCOVA analysis was carried out on n = 104 participants with NEI VFQ-25 data at both baseline and 26 weeks (Supplementary Table 6). There was no significant difference between the two groups. Similarly, a sensitivity analysis on n = 120 participants with data at baseline and with a visit within an 8-week window of the 26 weeks follow-up visit showed no significant difference between the groups. Estimated mean difference at 26 weeks taken from ANCOVA model, adjusting for baseline score and hemianopia type was −4.04 (95% confidence interval (CI): −9.45 to 1.36; p value: 0.141) for primary analysis and −2.33 (95% CI: −7.42 to 2.75; p value: 0.365) for sensitivity analysis. At 26 weeks, there was a 5.8 point difference between groups which was not clinically significantly different.

#### Secondary outcome analysis

Secondary outcome results are outlined in Supplementary Table 6; there were no significant differences between the two treatment groups for all secondary outcome measures. Sensitivity analysis for these secondary outcomes is further outlined in Supplementary files. The estimated mean difference for NEADL from a random effects longitudinal model was −0.5 (95% CI: −1.58 to 0.58; p value: 0.362). At 26 weeks, there was a 0.2 point difference between groups which was not clinically significantly different. For the EQ VAS (EQ Visual Analogue Scale—EQ-5D-5L), estimated mean difference from a random effects longitudinal model was 2.62 (95% CI: −2.28 to 7.51; p value: 0.293). At 26 weeks, there was a 1.3 point difference between groups which was not clinically significantly different.

The estimated mean difference for the BIVI-IQ from a random effects longitudinal model was 0.25 (95% CI: −0.82 to 1.33; p value: 0.645). At 26 weeks, there was a 1.3 point difference between groups which was not clinically significantly different. For extent of hemianopia, the estimated mean difference at 26 weeks taken from the ANCOVA model was 0.48 (95% CI: −4.54 to 5.5; p value: 0.85). For scanning accuracy and scanning speed, the estimated mean differences from a random effects longitudinal model were −2.64 (95% CI: −7.31 to 2.03; p value: 0.266) and 22.63 (95% CI: −6.56 to 51.82; p value: 0.128), respectively. At 26 weeks, there was a 2.6 point difference between groups for scanning accuracy which was not clinically significantly different. For scanning speed, at 26 weeks, there was a 0.4 point difference between groups which was not of clinical significance.

There were 29 (20 participants) related AEs in this trial and spread across both groups (Supplementary Table 7). Twenty-five were mild, two moderate, and two severe, and largely related to eye strain, headache, and blurred vision. There were five non-related SAEs which related to new strokes, high blood pressure and a fall with urinary tract infection.

## Discussion

This trial, comparing VST to sham training, demonstrated improvements in both groups across all primary and secondary outcome measures, with no significant differences between the two groups at 26 weeks. Baseline characteristics including age, sex, stroke type, laterality, severity, time since onset, visual acuity, and hemianopia type, were well-matched between groups. The primary outcome, the NEI VFQ-25 questionnaire, is a validated measure of vision-related quality of life commonly used in hemianopia studies.^20^ Secondary outcomes assessed activities of daily living, quality of life, and objective measures of scanning accuracy and speed.

Several factors may explain the improvements observed in both groups and the lack of a between-group difference, including the type of training, placebo effects, additional support provided, training dose and compliance, timing of intervention, and sample size.

### Type of training

VST, supported by evidence from a Cochrane review^12^ and the European Stroke Organization Vision guideline,^26^ is considered a promising compensatory intervention for hemianopia. Scanning of the environment relies on fast, saccadic eye movements, which are integral to compensatory visual training programs. Studies have shown that stroke survivors with hemianopia often exhibit disorganized scanning patterns, reduced saccadic amplitude, and slower target detection on the blind side.^13^ Interventions like VST aim to reorganize these scanning behaviors, leading to improved detection speed and accuracy. While our VST targeted these fast saccadic movements, the sham training used slow, tracking movements, which, though not standard for scanning, may have facilitated blind-field awareness and adaptive compensation. However, the broader translation of these improvements to activities of daily living and quality of life remains uncertain and warrants further exploration. Our VST training was designed to stimulate a 30° area of field of vision to the right and left sides—similar to many other computerized training programs. The training required the participant to make a series of voluntary saccades to either side of the central point, in a structured pattern, but without any time cut-off for finding each successive correct target and without any additional cues such as auditory signals. This differs to computer-generated tasks. It is possible that having a temporal imperative adds greater effect to visual scanning. RCTs of computer-generated visual scanning interventions have reported significant improvements in quality of life^25^ and in visual search performance.^27^ However, other trials have reported no significant improvement in search performance using computer-generated visual scanning therapy or virtual reality tasks.^28,29^ Further research is warranted to consider the added influence of time constraints during training, whether using computer-generated or paper-based tasks. A further issue with paper-based training may be the repetitive, unchanging demands of the training compared with computerized options which can alter the complexity and demands of training coupled with feedback on performance. While some participants may have found the training boring, the number of training hours achieved overall indicated good compliance with the paper-based training.

### Placebo effect

The placebo effect is a recognized factor in trials involving behavioral and compensatory training. Previous studies comparing VST to standard care or attention-based controls have also reported significant improvements in quality of life (NEI VFQ-25 outcomes (NEI VFQ-25 outcomes^15,25,27^). Sham training, such as attention tasks^28^ or audio-cued VST,^27^ often yields outcomes indistinguishable from active training. Similarly, our sham training, which involved slow pursuit movements into both hemifields, may have engaged adaptive mechanisms for compensatory awareness, effectively functioning as an active intervention.

### Additional support

Improvements in both groups may also reflect the impact of comprehensive support, including detailed patient information and clinical team involvement. The SEARCH trial provided participants with extensive informational resources, fulfilling a long-standing request from stroke survivors. This contrasts with the more limited information provided in earlier trials, such as the VISION pilot trial.^15^ The enhanced understanding of their condition may have empowered participants and contributed to their progress, independent of the training type.

### Training dose and compliance

Both groups demonstrated good compliance, with participants completing an average of 48 hours of VST and 31.4 hours of sham training. These training doses exceed those reported in many previous studies, where effectiveness often plateaued after 20 hours.^19,25,30,31^ Continued use of training in the VST group at 6 and 12 weeks suggests strong acceptability. However, the higher dose in the VST group may not have translated into significantly better outcomes, underscoring the potential contribution of non-specific factors in both interventions. Compliance information was taken from patient diaries which were self-reported and, hence, are subject to participant bias. However, we would expect this to balance between the two groups similar to the balance for all other outcome measures and reports.

### Timing of intervention

The trial included participants between 1 to 6 months post-stroke, a window often associated with stable hemianopia. Although some participants (13% VST, 18% sham training) experienced delayed recovery, these cases were evenly distributed and unlikely to have influenced the outcomes significantly. Recruitment timing and recovery phase alignment with other studies further validate the findings.

### Sample size

While the SEARCH trial surpassed the recruitment targets of previous hemianopia studies, loss to follow-up reduced the sample size for primary analysis. Nonetheless, sensitivity analyses achieved the necessary power (n = 120 participants) to detect an 8-point difference in NEI VFQ-25 scores. Retention challenges remain a common limitation in hemianopia trials, as highlighted by Aimola et al.^28^ where participant dropout due to lack of motivation was significant.

## Implications for clinical practice

These results do not exclude the potential value of VST as a treatment option for hemianopia. Improvements observed across both groups reinforce the importance of providing stroke survivors with timely information and supportive interventions. Consistent with patient and family requests, vision information resources should be made readily accessible following diagnosis, leveraging existing formats such as print and online materials (e.g. VISION Research Unit,^32^ British and Irish Orthoptic Society,^33^ The Stroke Association^34^ and Royal National Institute for Blind People^35^).

## Future research

Future research should address limitations in current evidence, including small sample sizes, ineffective placebos/controls, and lack of rigorous concealment. The apparent therapeutic impact of sham training warrants investigation into the differential effects of slow pursuit versus fast saccadic eye movements on compensatory mechanisms. Trials must also disentangle the contributions of psychosocial support and informational resources from the active effects of training interventions.

## Conclusion

The SEARCH trial compared an active intervention of fast eye movement VST versus sham training using slow tracking eye movements. Both groups demonstrated improvement across all primary and secondary outcomes with no significant differences observed between them. However, the potential influence of placebo effect driven by additional information resources and clinician input during the first 6 weeks of treatment cannot be excluded. Furthermore, the sham training may have contributed to improvements, functioning as an active intervention.

The findings raise important questions for future research. Both visual scanning and sham training were associated with improvements in quality of life, activities of daily living, and objective visual search function. The absence of a pure control group in this trial limits definitive conclusions regarding the extent of improvement attributable to training interventions alone.

These results should not be interpreted as a reason to exclude VST from the treatment options offered to stroke survivors with hemianopia. VST should continue to be provided, and its effects monitored, for individual stroke survivors. However, further research is required to disentangle the specific effects of active interventions from non-specific factors such as informational and psychosocial support. We emphasize the importance of providing comprehensive, vision-specific information to stroke survivors and their families as part of holistic care.

## Supplemental Material

sj-docx-1-wso-10.1177_17474930251330140 – Supplemental material for A randomized controlled trial of Scanning Eye trAining as a Rehabilitation Choice for Hemianopia after stroke (SEARCH) Supplemental material, sj-docx-1-wso-10.1177_17474930251330140 for A randomized controlled trial of Scanning Eye trAining as a Rehabilitation Choice for Hemianopia after stroke (SEARCH) by Fiona J Rowe, Ella Brayshaw, Michaela Brown, Kausik Chatterjee, Avril Drummond, Christine Hazelton, Brin Helliwell, Lauren R Hepworth, Claire Howard, Stevie Johnson, Carmel Noonan, Catherine Sackley and Laura Wright in International Journal of Stroke

sj-docx-2-wso-10.1177_17474930251330140 – Supplemental material for A randomized controlled trial of Scanning Eye trAining as a Rehabilitation Choice for Hemianopia after stroke (SEARCH) Supplemental material, sj-docx-2-wso-10.1177_17474930251330140 for A randomized controlled trial of Scanning Eye trAining as a Rehabilitation Choice for Hemianopia after stroke (SEARCH) by Fiona J Rowe, Ella Brayshaw, Michaela Brown, Kausik Chatterjee, Avril Drummond, Christine Hazelton, Brin Helliwell, Lauren R Hepworth, Claire Howard, Stevie Johnson, Carmel Noonan, Catherine Sackley and Laura Wright in International Journal of Stroke
