# Supplementary material for: A randomized controlled trial of Scanning Eye trAining as a Rehabilitation Choice for Hemianopia after stroke (SEARCH)
Source: Int J Stroke. 2025 Mar 13;20(8):968–76. doi: 10.1177/17474930251330140 (PMC12446690; doi:10.1177/17474930251330140)
Supplement: sj-docx-1-wso-10.1177_17474930251330140 – Supplemental material for A randomized controlled trial of Scanning Eye trAining as a Rehabilitation Choice for Hemianopia after stroke (SEARCH) [file sj-docx-1-wso-10.1177_17474930251330140.docx]

**Supplementary files**

**Supplementary Table 1, Reasons for ineligibility, non-consent and non-randomisation**

| **Ineligible reasons: overall n=2599 patients (2705 reasons)**  R1= Inability to undertake treatment, e.g. due to severe cognitive impairment  R2= Visual inattention to visual field impairment  R3= Other serious concomitant medical condition, e.g. palliative care  R4= Pre-existing visual field impairment due to previous stroke  R5= Not 18 years or over  R6= Not a clinically diagnosed stroke  R7= Not stable homonymous hemianopia (partial or complete) induced by stroke occurring ≥4 weeks ago but ≤26 weeks ago  R8= Not able to undertake treatment  R9= Unable to consent/understand study  R10= Met exclusion or did not meet inclusion - no further reason given | **N (%)**  245 (9.4)  470 (18.1)  96 (3.7)  113 (4.3)  5 (0.2)  89 (3.4)  1548 (59.6)  39 (1.5)  66 (2.5)  34 (1.3) |
| --- | --- |
| **Non-consent reasons: overall n=110**  R1= Did not provide reason  R2= Did not wish to fill out questionnaires/diaries  R3= Did not want to be randomly assigned treatment  R4= Did not want to be assigned to information only group  R5= Did not want to attend follow up visits  R6= Other | **N (%)**  41 (37.3)  9 (8.2)  4 (3.6)  1 (0.9)  36 (32.7)  19 (17.3) |
| **Non-randomisation reasons: overall n=999**  R1= Further stroke  R2= Further TIA  R3= Died  R4= Unable to contact patient to arrange baseline visit  R5= Could not attend follow-up visits  R6= Moved area  R7= Did not attend baseline appointment  R8= Could not attend baseline appointment  R9= Other | **N (%)**  10 (1)  0 (0)  175 (17.5)  26 (2.6)  34 (3.4)  15 (1.5)  51 (5.1)  136 (13.6)  552 (55.3) |

**Supplementary Table 2, Protocol deviations**

|  | **VST** | | **SHAM** | | **Total** | |
| --- | --- | --- | --- | --- | --- | --- |
| **Protocol Deviations:** | **Events, n** | **Participants n (%)** | **Events, n** | **Participants n (%)** | **Events,**  **n** | **Participants n (%)** |
| Any protocol deviation | 16 | 12 (15.4%) | 17 | 15 (18.8%) | 33 | 27 (17.1%) |
| Deviations detailed separately: |  |  |  |  |  |  |
| Assessments/questionnaires not carried out at visit | 4 | 4 (5.1%) | 6 | 6 (7.5%) | 10 | 10 (6.3%) |
| Original copy of questionnaire not in file | 1 | 1 (1.3%) | 0 | 0 | 1 | 1 (0.6%) |
| Original copy of questionnaire not in file and missing diary | 1 | 1 (1.3%) | 0 | 0 | 1 | 1 (0.6%) |
| Patient not given treatment or diary at baseline visit | 1 | 1 (1.3%) | 0 | 0 | 1 | 1 (0.6%) |
| Patient who had previous scanning training is randomised to the study | 1 | 1 (1.3%) | 0 | 0 | 1 | 1 (0.6%) |
| Randomised before baseline assessment | 0 | 0 | 1 | 1 (1.3%) | 1 | 1 (0.6%) |
| Spontaneous recovery of hemianopia after randomisation | 0 | 0 | 1 | 1 (1.3%) | 1 | 1 (0.6%) |
| Stroke occurred <4 OR >26 weeks ago | 0 | 0 | 1 | 1 (1.3%) | 1 | 1 (0.6%) |
| Telephone log not completed | 1 | 1 (1.3%) | 1 | 1 (1.3%) | 2 | 2 (1.3%) |
| Visits not carried out as per protocol | 1 | 1 (1.3%) | 1 | 1 (1.3%) | 2 | 2 (1.3%) |
| Wrong strata selected at randomisation | 6 | 6 (7.7%) | 6 | 6 (7.5%) | 12 | 12 (7.6%) |

**Supplementary Table 3, Baseline demographics**

|  | **VST**  **N=78** | **SHAM**  **N=80** | **Total**  **N=158** |
| --- | --- | --- | --- |
| Age, Years: Mean (SD) | 66.6 (13.6) | 64.9 (14.3) | 65.8 (14.0) |
| Sex : n(%) |  |  |  |
| Female | 23 (29.5%) | 21 (26.3%) | 44 (27.8%) |
| Male | 55 (70.5%) | 59 (73.8%) | 114 (72.2%) |
| Ethnicity : n(%) |  |  |  |
| White | 74 (94.9%) | 75 (93.8%) | 149 (94.3%) |
| Black or Black British | 0 | 3 (3.8%) | 3 (1.9%) |
| Asian or Asian British | 2 (2.6%) | 2 (2.5%) | 4 (2.5%) |
| Other : African | 1 (1.3%) | 0 | 1 (0.6%) |
| Other : British Arab | 1 (1.3%) | 0 | 1 (0.6%) |
| Stroke Type: n(%) |  |  |  |
| Ischaemic | 74 (94.9%) | 71 (88.8%) | 145 (91.8%) |
| Haemorrhagic | 4 (5.1%) | 9 (11.3%) | 13 (8.2%) |
| Thrombolysis: n(%) |  |  |  |
| Yes | 3 (3.8%)^A^ | 8 (10%)^A^ | 11 (7%)^A^ |
| No | 74 (94.9%)^A^ | 70 (87.5%)^A^ | 144 (91.1%)^A^ |
| Side of Infarct: n(%) |  |  |  |
| Left | 39 (50%) | 45 (56.3%) | 84 (53.2%) |
| Right | 36 (46.2%) | 32 (40%) | 68 (43%) |
| Bilateral | 3 (3.8%) | 3 (3.8%) | 6 (3.8%) |
| Area of Brain Affected: n(%) |  |  |  |
| Frontal Lobe | 4 (5.1%) | 2 (2.5%) | 6 (3.8%) |
| Parietal Lobe | 8 (10.3%) | 13 (16.3%) | 21 (13.3%) |
| Temporal Lobe | 6 (7.7%) | 7 (8.8%) | 13 (8.2%) |
| Occipital Lobe | 70 (89.7%) | 68 (85%) | 138 (87.3%) |
| Cerebellum | 1 (1.3%) | 3 (3.8%) | 4 (2.5%) |
| Thalamus | 6 (7.7%) | 4 (5%) | 10 (6.3%) |
| Basal Ganglia | 2 (2.6%) | 1 (1.3%) | 3 (1.9%) |
| Brainstem | 1 (1.3%) | 1 (1.3%) | 2 (1.3%) |
| Capsule | 0 | 2 (2.5%) | 2 (1.3%) |
| Other | 6 (7.7%) | 9 (11.3%) | 15 (9.5%) |
| Time since stroke onset (Days): Median (LQ, UQ) | 92.0 (57.0,134.0) | 88.5 (70.0,132.5) | 90.5 (66.0,133.0) |
| Barthel Index Total score: Mean (SD) | 18.9 (2.9) | 19.0 (2.0) | 19.0 (2.5) |
| **Symptoms** |  |  |  |
| Diplopia | 6 (7.7%) | 3 (3.8%) | 9 (5.7%) |
| Blurred/Reduced Vision | 25 (32.1%)^B^ | 21 (26.3%) | 46 (29.1%)^B^ |
| Reading Difficulties | 37 (47.4%) | 39 (48.8%) | 76 (48.1%) |
| Visual Field Loss - Right | 39 (50%) | 46 (57.5%) | 85 (53.8%) |
| Visual Field Loss - Left | 37 (47.4%)^B^ | 33 (41.3%) | 70 (44.3%)^B^ |
| Oscillopsia | 3 (3.8%) | 0 | 3 (1.9%) |
| Perceptual Difficulties | 21 (26.9%) | 17 (21.3%)^C^ | 38 (24.1%)^C^ |
| Other | 2 (2.6%)^D^ | 4 (5%) ^D^ | 6 (3.8%) ^D^ |

^A^ Missing or one participant in VST group and 2 participants in the sham group

^B^ Missing for one participant in the VST group

^C^ Missing for one participant in the sham group

^D^ Missing for three participants in the VST group and four participants in the sham group

**Supplementary Table 4, Baseline visual measurements**

|  | **VST** | **SHAM** | **Total** |
| --- | --- | --- | --- |
| Best Corrected Visual Acuity (LogMAR) | **N=73** | **N=75** | **N=148** |
| Near Left |  |  |  |
| Mean (SD) | 0.3 (0.2) | 0.2 (0.2) | 0.2 (0.2) |
| Near Right |  |  |  |
| Mean (SD) | 0.2 (0.2) | 0.2 (0.2) | 0.2 (0.2) |
| Distance Left | **N=78** | **N=80** | **N=158** |
| Mean (SD) | 0.2 (0.2) | 0.1 (0.2) | 0.2 (0.2) |
| Distance Right |  |  |  |
| Mean (SD) | 0.2 (0.2) | 0.1 (0.2) | 0.1 (0.2) |
| Standard Cancellation Test Duration (Seconds) | **N=76** | **N=79** | **N=155** |
| Mean (SD) | 29.4 (18.2) | 32.4 (17.4) | 30.9 (17.8) |
| Number of Clocks Crossed Out | **N=78** | **N=80** | **N=158** |
| Mean (SD) | 47.8 (5.2) | 49.1 (4.4) | 48.4 (4.8) |
| Ocular Motility: | **N=78** | **N=80** | **N=158** |
| Normal | 69 (88.5%) | 77 (96.3%) | 146 (92.4%) |
| Abnormal | 8 (10.3%) | 3 (3.8%) | 11 (7%) |
| Near Point of Convergence (cm) | **N=77** | **N=80** | **N=157** |
| Mean (SD) | 8.1 (2.6) | 7.6 (2.9) | 7.8 (2.7) |
| Visual Field Assessment Diagnosis: | **(N = 78)** | **(N = 80)** | **(N = 158)** |
| Homonymous Hemianopia - Left - Partial | 34 (43.6%) | 25 (31.3%) | 59 (37.3%) |
| Homonymous Hemianopia - Right - Partial | 28 (35.9%) | 39 (48.8%) | 67 (42.4%) |
| Homonymous Hemianopia - Left - Complete | 3 (3.8%) | 8 (10%) | 11 (7%) |
| Homonymous Hemianopia - Right - Complete | 13 (16.7%) | 8 (10%) | 21 (13.3%) |

^A^ Missing for 22 participants in the VST group and 29 participants in the sham group

^B^ Missing for 19 participants in the VST group and 26 participants in the sham group

**Supplementary Table 5, Treatment hours within first 6 weeks and overall**

|  | **VST**  **N=78** | **SHAM**  **N=80** |
| --- | --- | --- |
| Treatment hours within first 6 Weeks: Median (LQ, UQ) | 18.0 (15.2,20.5)^A^ | 16.4 (9.8,19.5)^A^ |
| Treatment hours Overall: Median (LQ, UQ) | 35.0 (20.5,71.3)^B^ | 20.5 (14.0,42.2)^B^ |

**Supplementary Table 6, Primary analysis – NEI VFQ-25 quality of life questionnaire scores.**

***A higher score indicates a higher quality of life***

|  | VST | | Sham | | Estimated mean difference  (95%) confidence interval | p-value |
| --- | --- | --- | --- | --- | --- | --- |
|  | Baseline  Mean (SD) | 26 week follow-up  Mean (SD) | Baseline  Mean (SD) | 26 week follow-up  Mean (SD) |  |  |
| Primary outcome | | | | | | |
| NEI VFQ-25* | 54.4 (17.4) | 58.3 (20) | 54.6 (20) | 64.1 (19.3) | -4.04 (-9.45, 1.36) | 0.141 |
| NEI VFQ-25 Sensitivity analysis* | 54.4 (17.4) | 58.8 (20.2) | 54.6 (20) | 61.8 (20.9) | -2.33 (-7.42, 2.75) | 0.365 |
| Secondary outcomes | | | | | | |
| NEADL** | 14.8 (5.8) | 16.3 (5) | 13.9 (6.5) | 16.1 (5.2) | -0.5 (-1.58, 0.58) | 0.362 |
| EQ-VAS** | 64.6 (20.6) | 68.1 (20.4) | 64.5 (20.7) | 69.4 (20.2) | 2.62 (-2.28, 7.51) | 0.293 |
| BIVI-IQ* | 14.3 (5.5) | 13 (6.2) | 13.3 (6.3) | 11.7 (5.4) | 0.25 (-0.82 , 1.33) | 0.645 |
| Percentage of VF affected* | 76 (19.4) | 69.7 (20.7) | 79.4 (18.1) | 71.4 (23.3) | 0.48 (-4.54, 5.5) | 0.85 |
| Percentage correct object identification** | 69.6 (16.4) | 76.7 (18.1) | 70.8 (16.9) | 79.3 (16.9) | -2.64 (-7.31, 2.03) | 0.266 |
| Speed of identification (seconds)** | 94.2 (61.2) | 91.5 (47.8) | 104.6(62) | 95.1 (54.7) | 22.63 (-6.56, 51.82) | 0.128 |

* Estimated mean difference from ANCOVA model adjusting for baseline score/measurement treatment group and partial/complete hemianopia

** Estimated mean difference from repeated measures mixed effects model adjusting for baseline score/measurement, treatment group, timepoint and partial/complete hemianopia.

**Supplementary Table 7, Related adverse events by severity**

|  | | **VST** | | **SHAM** | | **Total** | |
| --- | --- | --- | --- | --- | --- | --- | --- |
| **Non-Serious adverse event** | **Severity** | **Events n** | **Patients n(%)** | **Events n** | **Patients n(%)** | **Events n** | **Patients n(%)** |
| Eyestrain | Mild | 6 | 5 (6.4%) | 4 | 4 (5%) | 10 | 9 (5.7%) |
|  | Severe | 1 | 1 (1.3%) | 0 | 0 | 1 | 1 (0.6%) |
| Fatigue | Mild | 0 | 0 | 1 | 1 (1.3%) | 1 | 1 (0.6%) |
| Headache | Mild | 4 | 2 (2.6%) | 2 | 2 (2.5%) | 6 | 4 (2.5%) |
|  | Moderate | 1 | 1 (1.3%) | 1 | 1 (1.3%) | 2 | 2 (1.3%) |
|  | Severe | 1 | 1 (1.3%) | 0 | 0 | 1 | 1 (0.6%) |
| Other: Aching eyes | Mild | 0 | 0 | 1 | 1 (1.3%) | 1 | 1 (0.6%) |
| Other: Blurred vision | Mild | 1 | 1 (1.3%) | 0 | 0 | 1 | 1 (0.6%) |
| Other: Blurred vision and headache | Mild | 1 | 1 (1.3%) | 0 | 0 | 1 | 1 (0.6%) |
| Other: Burning | Mild | 1 | 1 (1.3%) | 0 | 0 | 1 | 1 (0.6%) |
| Other: Burning sensation in eyes | Mild | 0 | 0 | 1 | 1 (1.3%) | 1 | 1 (0.6%) |
| Other: Motion Sickness | Mild | 1 | 1 (1.3%) | 0 | 0 | 1 | 1 (0.6%) |
| Other: Seeing flashing lights | Mild | 1 | 1 (1.3%) | 0 | 0 | 1 | 1 (0.6%) |
| Other: Stiff Neck | Mild | 0 | 0 | 1 | 1 (1.3%) | 1 | 1 (0.6%) |
| **Total** | **Mild** | **15** | **10 (12.8%)** | **10** | **7 (8.8%)** | **25** | **17 (10.8%)** |
|  | **Moderate** | **1** | **1 (1.3%)** | **1** | **1 (1.3%)** | **2** | **2 (1.3%)** |
|  | **Severe** | **2** | **1 (1.3%)** | **0** | **0** | **2** | **1 (0.6%)** |
| **Serious adverse event – unrelated** | **Severity** | **Events n** | **Patients n(%)** | **Events n** | **Patients n(%)** | **Events n** | **Patients n(%)** |
| New stroke – in-patient hospitalisation | Mild | 1 | 1 (1.3%) | 0 | 0 | 1 | 1 (1.3%) |
|  | Moderate | 0 | 0 | 1 | 1 | 1 | 1 (1.3%) |
|  | Severe | 1 | 1 (1.3%) | 0 | 0 | 1 | 1 (1.3%) |
| High blood pressure – in-patient hospitalisation | Mild | 1 | 1 (1.3%) | 0 | 0 | 1 | 1 (1.3%) |
| Fall with urinary tract infection | Severe | 1 | 1 (1.3%) | 0 | 0 | 1 | 1 (1.3%) |

*Note: Where patients have experienced more than one adverse event and more than one severity, they have been reported in the most severe category.*
