# Supplementary material for: A randomized controlled trial of Scanning Eye trAining as a Rehabilitation Choice for Hemianopia after stroke (SEARCH)
Source: Int J Stroke. 2025 Mar 13;20(8):968–76. doi: 10.1177/17474930251330140 (PMC12446690; doi:10.1177/17474930251330140)
Supplement: sj-docx-2-wso-10.1177_17474930251330140 – Supplemental material for A randomized controlled trial of Scanning Eye trAining as a Rehabilitation Choice for Hemianopia after stroke (SEARCH) [file sj-docx-2-wso-10.1177_17474930251330140.docx]

**Supplementary Figure 1: Intervention**


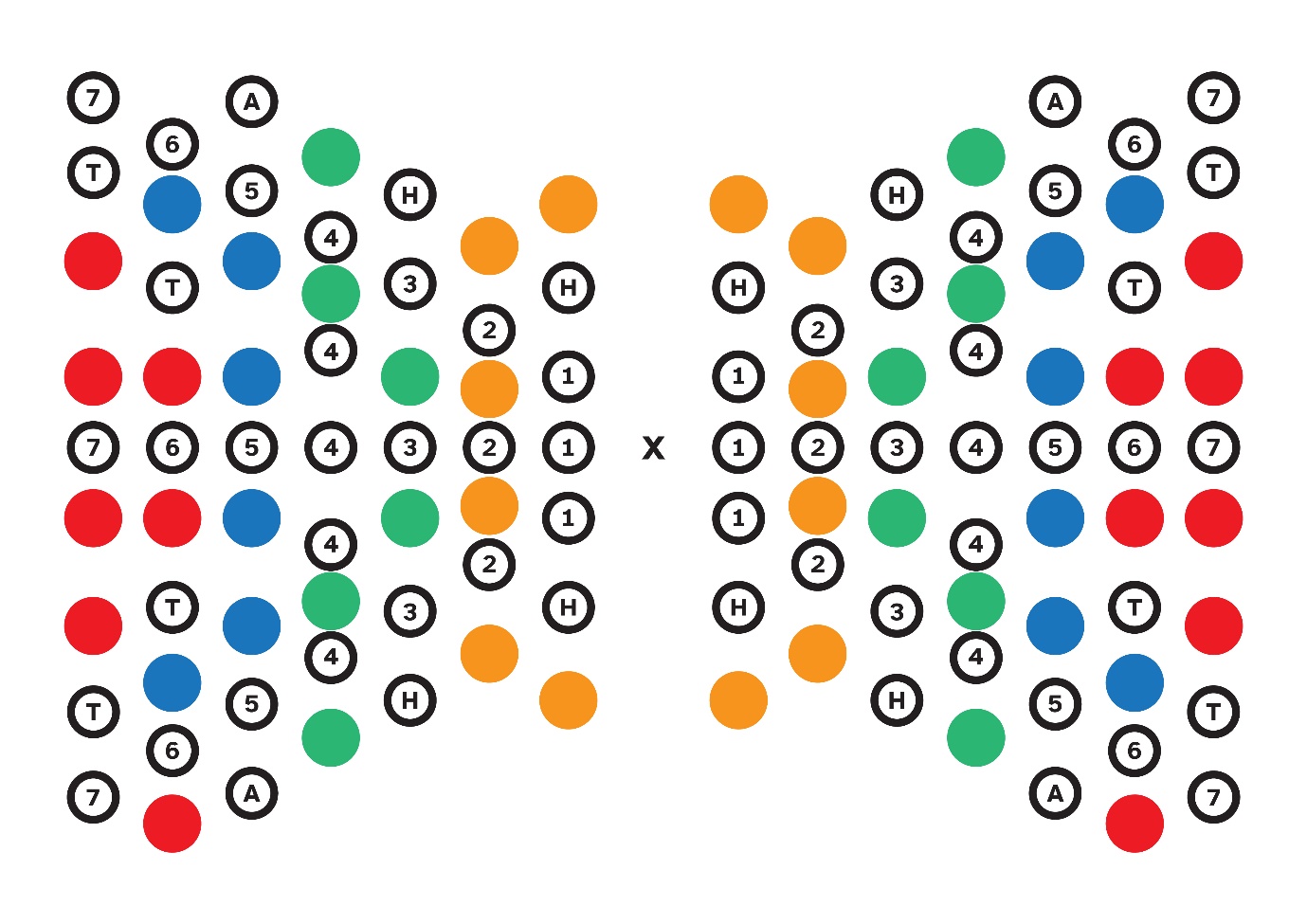


Legend: A4 training card held close to eyes.

The patient held the sheet at a distance of 8 inches/20cms from their eyes to achieve a 30-degree area of field of vision to the right and left sides. Following the instruction sheet, the patient performed a series of tasks, in which they transferred/jumped gaze quickly between targets. Tasks included detection of and discrimination between different targets. This engaged saccadic (fast) eye movements required for scanning and search processes. Coloured targets could be changed to patterned targets if the patient had a colour vision deficiency. The training period could run beyond 6 weeks if the participant did not gain the minimum treatment dose (20 hours) over 6 weeks. The participant looks quickly between targets during tasks including detection (find the target) and discrimination (find specified targets – colours/numbers). The image above outlines, through the sequence of blue arrows, a discrimination task to find increasing numbers (1 to 7) on horizontal scanning to right-left sides. Presbyopia is considered. Reading glasses could be used. However, as the targets are coloured and of large size, they are easily seen at near without the need for reading glasses by most participants.

**Supplementary Figure 2, Final adjusted recruitment graph**


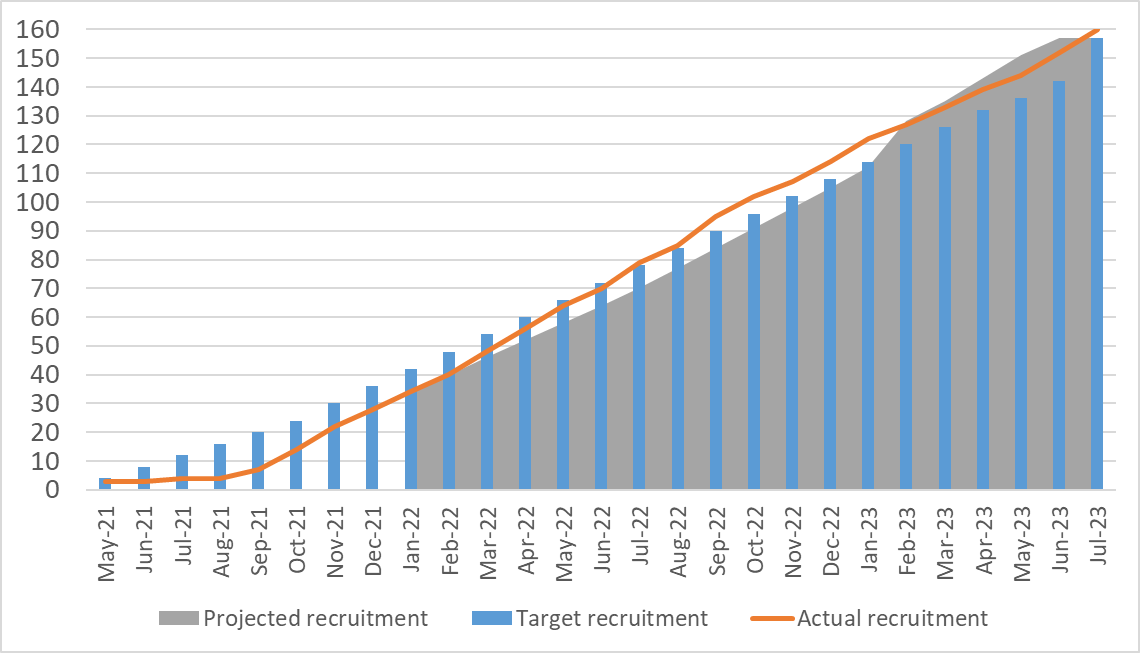


**Supplementary Figure 3, CONSORT flow diagram**

- Included in Intention to Treat (n= 78)
- Included in primary analysis (n= 54)
  - Not in primary analysis but in sensitivity (n= 8)
  - Insufficient data for either primary or sensitivity (n= 16)

Allocated to VST (n= 78)

- Received allocated intervention (n= 78)
- Did not received allocated intervention (n= 0)

Allocated to Sham Training (n= 80)

- Received allocated intervention (n= 80)
- Did not received allocated intervention (n= 0)
- Included in Intention to Treat (n= 80)
- Included in primary analysis (n= 50)
  - Not in primary analysis but in sensitivity (n= 8)
  - Insufficient data for either primary or sensitivity (n= 22)

Excluded (n= 3708)

- Not meeting eligibility criteria (n= 2599)
- Declined consent (n= 110)
- Other reasons (n= 999)

Assessed for eligibility

(n= 3869)

Randomised

(n= 161)*

Lost to follow-up (n= 4)

- Patient did not attend follow-up visits (n= 1)
- Unable to contact (n= 1)
- Patient unwell (n= 1)
- Patient decision (n= 1)

Lost to follow-up (n= 7)

- Patient did not attend follow-up visits (n= 1)
- Unable to contact (n= 6)

Follow-up

Analysis

Allocation

Enrolment

*3 of the 161 patients randomised withdrew consent to use data**.**
